# Identification of Novel Trypanosoma cruzi Proteasome Inhibitors Using a Luminescence-Based High-Throughput Screening Assay

**DOI:** 10.1128/AAC.00309-19

**Published:** 2019-08-23

**Authors:** Filip Zmuda, Lalitha Sastry, Sharon M. Shepherd, Deuan Jones, Alison Scott, Peter D. Craggs, Alvaro Cortes, David W. Gray, Leah S. Torrie, Manu De Rycker

**Affiliations:** a Drug Discovery Unit, Wellcome Centre for Anti-Infectives Research, School of Life Sciences, University of Dundee, Dundee, United Kingdom; b Protein Production Team, Wellcome Centre for Anti-Infectives Research, School of Life Sciences, University of Dundee, Dundee, United Kingdom; c Screening Compound Profiling and Mechanistic Biology, Platform Technology and Science, GlaxoSmithKline, Stevenage, United Kingdom

**Keywords:** Chagas’ disease, *Trypanosoma cruzi*, assay development, drug discovery, drug screening, pharmacology, proteasome

## Abstract

Chagas’ disease, caused by the protozoan parasite Trypanosoma cruzi, is a potentially life-threatening condition that has become a global issue. Current treatment is limited to two medicines that require prolonged dosing and are associated with multiple side effects, which often lead to treatment discontinuation and failure. One way to address these shortcomings is through target-based drug discovery on validated T. cruzi protein targets. One such target is the proteasome, which plays a crucial role in protein degradation and turnover through chymotrypsin-, trypsin-, and caspase-like catalytic activities. In order to initiate a proteasome drug discovery program, we isolated proteasomes from T. cruzi epimastigotes and characterized their activity using a commercially available glow-like luminescence-based assay. We developed a high-throughput biochemical assay for the chymotrypsin-like activity of the T. cruzi proteasome, which was found to be sensitive, specific, and robust but prone to luminescence technology interference. To mitigate this, we also developed a counterscreen assay that identifies potential interferers at the levels of both the luciferase enzyme reporter and the mechanism responsible for a glow-like response. Interestingly, we also found that the peptide substrate for chymotrypsin-like proteasome activity was not specific and was likely partially turned over by other catalytic sites of the protein. Finally, we utilized these biochemical tools to screen 18,098 compounds, exploring diverse drug-like chemical space, which allowed us to identify 39 hits that were active in the primary screening assay and inactive in the counterscreen assay.

## Abstract (precis)

Chagas’ disease, caused by the protozoan parasite Trypanosoma cruzi, is a potentially life-threatening condition that has become a global issue. Current treatment is limited to two medicines that require prolonged dosing and are associated with multiple side effects, which often lead to treatment discontinuation and failure. One way to address these shortcomings is through target-based drug discovery on validated T. cruzi protein targets.

## TEXT

Chagas’ disease is a parasitic disease caused by the kinetoplastid parasite Trypanosoma cruzi. The disease is a problem, not only in the regions of endemicity in Latin America, but also more globally because of migration (1, 2). Disease progression is characterized by an initial acute phase, with symptoms such as fever and local inflammation, followed by a long, symptomless indeterminate phase. In a subset of people, the disease develops into a symptomatic, chronic phase with cardiomyopathy and mega-organ disease as the main manifestations. Approximately 2% of infected people develop cardiac problems annually (3), with an associated death toll of around 10,000 per year (2). Treatment for Chagas’ disease is currently limited to the two nitroheterocyclic drugs benznidazole and nifurtimox. Benznidazole is typically used as front-line treatment, as it is better tolerated than nifurtimox, notwithstanding a 10% treatment discontinuation rate due to its own side effects (4). New, better-tolerated medicines are urgently required, but their development has proven very difficult, as exemplified by the failure in clinical trials of the only two new candidate treatments, posaconazole and fos-ravuconazole (5, 6). Many efforts are ongoing to identify new starting points for drug discovery, often through large-scale phenotypic screening (7–12). Target-based screening, where a particular protein is assayed in its purified state, is an alternative approach with the advantages of more straightforward understanding of chemical structure-activity relationships (SAR) due to the absence of cell membranes, a direct relationship between compound affinity and target inhibition or binding, and the opportunity to generate structural information to guide chemistry design. Lack of translation of target inhibition to parasite death is an important risk when investing in such a program, and selecting well-validated targets is essential. One powerful method to identify suitable protein targets is by determining the modes of action of compounds that show the desired phenotypic effects in terms of parasite killing. Recently, the proteasome was identified as a promising drug target for kinetoplastid diseases through mode-of-action determination of promising phenotypically active compounds (GNF6702 and GSK3494245/DDD01305143) (9, 13). The proteasome is a key component of the ubiquitin-proteasome protein degradation system and plays an important role in many cellular processes, including protein turnover and cell signaling (14). In eukaryotes, the proteasome comprises a central 20S cylindrical structure and two regulatory 19S complexes on either end of the 20S core. The 20S unit is made up of two outer (α) and two inner (β) polypeptide rings, where three of the β-type subunits are involved in chymotrypsin-, trypsin-, and caspase-like catalytic activities (15, 16). The proteasome is a well-exploited target in drug discovery for a variety of indications, including cancer, inflammation, and infectious diseases (17). In terms of parasitic diseases, the *Plasmodium* proteasome is well characterized, and proof of concept that selective inhibition is possible has opened the route to development of new malaria drugs targeting the proteasome (18). GNF6702 is active against Leishmania donovani, Trypanosoma brucei, and T. cruzi both *in vitro* and *in vivo* while showing no toxicity against mammalian cells, and GSK3494245/DDD01305143 is a preclinical candidate for visceral leishmaniasis developed from a T. cruzi screening hit, demonstrating that the proteasome is a suitable drug target across the kinetoplastid parasites. These compounds exert their effects on the parasites through the selective inhibition of the chymotrypsin-like activity of the parasite proteasome, and not the caspase- or trypsin-like activity (9, 13).

Attrition in drug discovery programs is high, and even compounds that demonstrate proof-of-concept efficacy in animal models frequently fail at later stages in the drug development process, often for non-target-related reasons (19). Once a validated target has been identified, it is therefore sensible to generate multiple chemical classes of inhibitors. With this in mind, we have started a hit discovery program for the T. cruzi proteasome chymotrypsin-like activity. Here, we present the development of a luminescence-based high-throughput screening (HTS) assay using partially purified T. cruzi proteasomes, as well as a technology interference counterscreen assay, which we then used to screen two diverse sets of compounds (18,098 compounds in total) in an effort to identify potential new starting points for a drug discovery program against Chagas’ disease.

## RESULTS AND DISCUSSION

### T. cruzi proteasome characterization.

**FIG 1:**
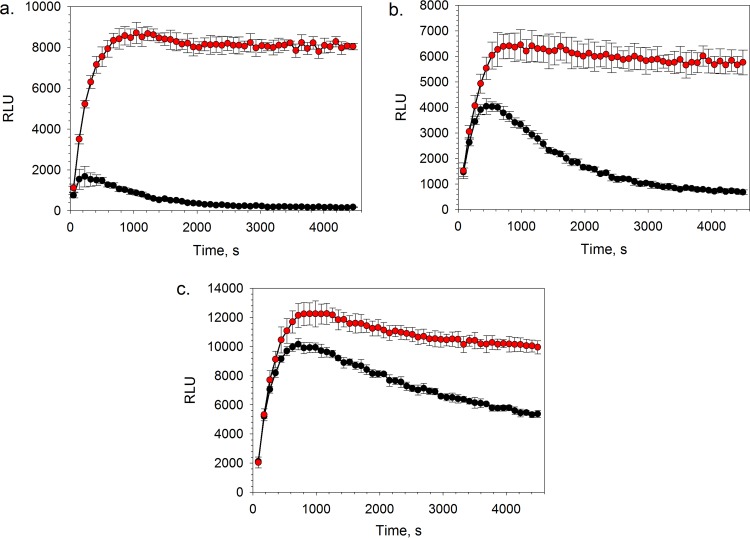
Chymotrypsin-like (a), trypsin-like (b), and caspase-like (c) activities of the T. cruzi proteasome in the presence (black circles) and absence (red circles) of 5 μM epoxomicin. Data are shown for 6 technical replicates (*n* = 6); the error bars represent standard deviations (SD).

Proteasomes were harvested from cleared epimastigote lysates through ultracentrifugation, followed by partial purification using size exclusion chromatography. In order to confirm the presence of enzymatic activity, chymotrypsin-, trypsin-, and caspase-like activities of the proteasome were measured using luminogenic versions of established peptide substrates (16, 20) and a commercially available glow response luminescence-based assay system (20). All three types of catalytic activities were found to be present in the pooled, partially purified T. cruzi proteasome material. To further profile the isolated protein, the catalytic activities of the proteasome were measured over time in the presence of the irreversible proteasome inhibitor epoxomicin (21, 22). The degree of inhibition of the chymotrypsin-like activity was the highest, followed by trypsin- and caspase-like activities (Fig. 1), which is consistent with previous literature reports (22). The characteristic plateau of the kinetic curves in the absence of inhibitor compound corresponded to steady-state conditions, where the rate of substrate consumption by the proteasome was equal to the rate of product consumption by the luciferase reporter enzyme (20). In the case of chymotrypsin-like activity, steady-state conditions were established within approximately 15 min and were maintained for the remainder of the 75-min kinetic experiment.

### High-throughput primary screening assay development.

**FIG 2:**
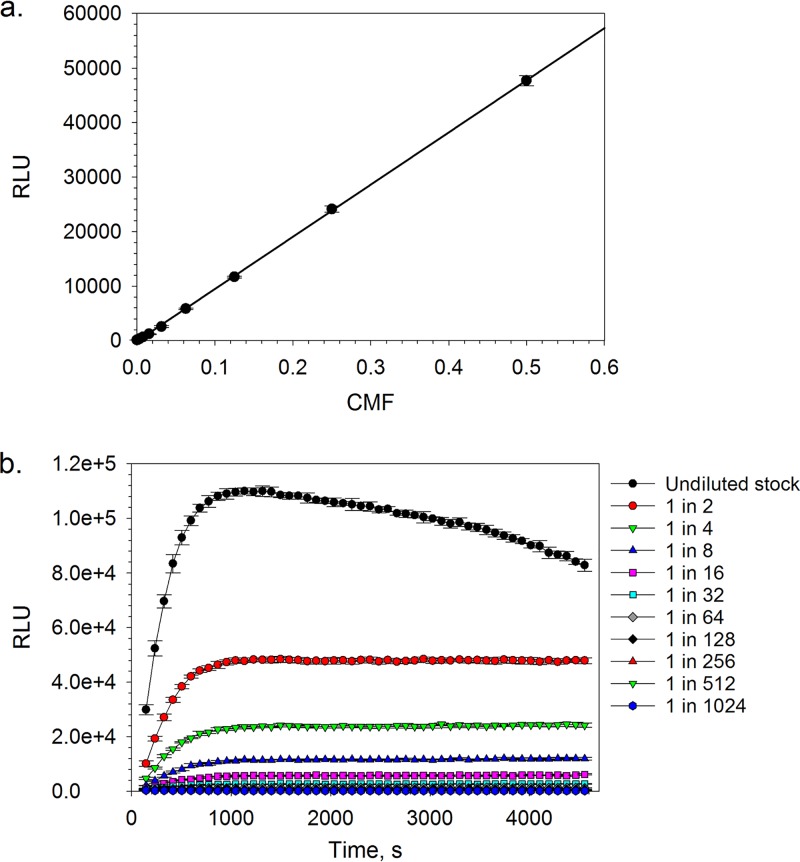
Chymotrypsin-like activity plotted as a function of either T. cruzi proteasome concentration after a 60-min biochemical reaction (a) or time at different proteasome stock solution dilutions (b). Data are shown for 5 technical replicates (*n* = 5); the error bars represent SD. Linear regression, *R*^2^ = 0.999.

**FIG 3:**
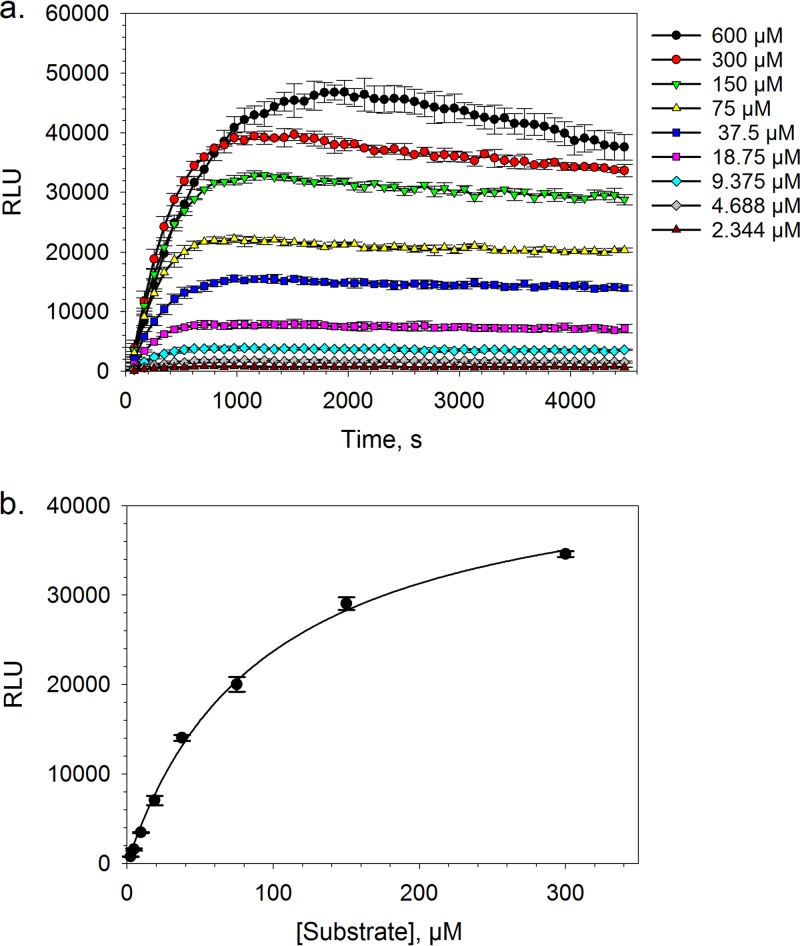
T. cruzi proteasome chymotrypsin-like activity plotted as a function of time in the presence of various concentrations of suc-Leu-Leu-Val-Tyr-aminoluciferin substrate (a) or as a function of the suc-Leu-Leu-Val-Tyr-aminoluciferin substrate concentration after a 60-min biochemical reaction fitted to the Michaelis-Menten model (apparent *K*_m_ = 93.5 μM [95% CI = 78.8 to 108.4 μM]) (b). Data are shown for 3 technical replicates (*n* = 3); the error bars represent SD.

Following the validation of the T. cruzi proteasome purification and isolation methodology, we diverted our efforts toward the optimization of the commercially available proteasome chymotrypsin-like activity luminescence-based assay for HTS. In the presence of a fixed amount of chymotrypsin-like substrate (i.e., succinyl-Leu-Leu-Val-Tyr-aminoluciferin) and under steady-state conditions, the luminescence response was shown to be linearly proportional (*R*^2^ = 0.9998) to the amount of T. cruzi proteasome up to a top concentration equivalent to a 1-in-2 dilution of the stock material (i.e., a concentration multiplication factor [CMF] of 0.5) (Fig. 2a). Steady-state conditions were established rapidly for all of the tested concentrations of the T. cruzi proteasome and were maintained throughout the course of the kinetic experiment, with the exception of the top protein concentration (i.e., undiluted stock), where a drop-off in the luminescence response was observed after approximately 30 min (Fig. 2b). This loss of steady state was likely a consequence of substrate depletion. By varying the concentration of suc-Leu-Leu-Val-Tyr-aminoluciferin in the presence of a fixed amount of T. cruzi proteasome, substrate inhibition was observed at a concentration of 600 μM under pre-steady-state conditions (Fig. 3a). This is in line with the work of Stein et al., who also reported proteasome chymotrypsin-like activity inhibition at high concentrations of a fluorescence-tagged variant of the suc-Leu-Leu-Val-Tyr peptide substrate (i.e., suc-Leu-Leu-Val-Tyr-AMC) (23). Utilizing the Z factor as a measure of assay quality, where values of ≥0.5 are generally accepted as sufficient for HTS (24), a 1-in-8 dilution (CMF = 0.125) of the stock T. cruzi proteasome preparation and a suc-Leu-Leu-Val-Tyr-aminoluciferin substrate concentration of 20 μM (final assay concentrations) were found to be optimal, affording Z factor values of >0.75 at steady state. Under these assay conditions, the apparent steady-state *K_m_* for the suc-Leu-Leu-Val-Tyr-aminoluciferin substrate was found to be 93.5 μM (95% confidence interval [CI] = 78.8 to 108.4 μM) (Fig. 3b), which was only marginally higher than the approximate value of 60 μM that was reported by O’Brien et al. using a similar assay platform in a cellular system (20). Finally, as our screening compound libraries are formulated in dimethyl sulfoxide (DMSO), the tolerance of the biochemical assay for this solvent was investigated. We found that the maximum tested concentration of DMSO (1% [vol/vol] final assay concentration) was well tolerated by the system and had a negligible effect on the luminescence response (see Fig. S2 in the supplemental material).

### High-throughput primary screening assay validation.

**FIG 4:**
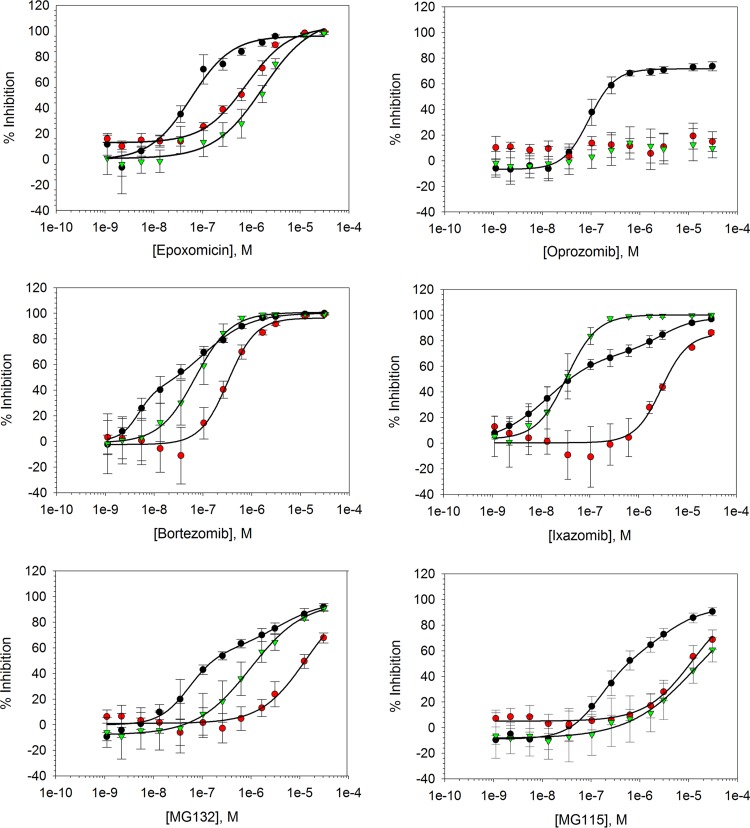
Cell-free T. cruzi proteasome chymotrypsin-like (black circles), trypsin-like (red circles), and caspase-like (green triangles) activity concentration-response curves for a panel of commercially available proteasome inhibitors (i.e., epoxomicin, oprozomib, bortezomib, ixazomib, MG132, and MG115). Data are shown for 4 independent replicates (*n* = 4). The error bars represent SD.

In order to validate the biochemical assay and identify a suitable control inhibitor compound for HTS, chymotrypsin-, trypsin-, and caspase-like activity concentration-response relationships for a panel of commercially available proteasome inhibitors were established (Fig. 4; see Table S1 in the supplemental material). Of the tested compounds, oprozomib exhibited specificity for the T. cruzi proteasome chymotrypsin-like active sites, which is in line with previous literature reports (9, 25). Interestingly, the compound failed to completely abolish catalytic activity in this biochemical assay. It was hypothesized that the suc-Leu-Leu-Val-Tyr-aminoluciferin substrate was not specific for the chymotrypsin-like active site of the T. cruzi proteasome, and residual turnover of the substrate by the trypsin- and/or caspase-like active sites resulted in incomplete apparent inhibition of chymotrypsin-like activity. To investigate this further, steady-state chymotrypsin-like activity concentration-response relationships were established for oprozomib using 20 μM and 600 μM suc-Leu-Leu-Val-Tyr-aminoluciferin substrate. It was envisaged that in the presence of a specific chymotrypsin-like activity inhibitor, an increase in nonspecific luminogenic substrate would result in an increased residual luminescence response and, subsequently, a reduced upper concentration-response curve plateau. The marked reduction in the upper concentration-response curve plateaus following a 30-fold increase in the suc-Leu-Leu-Val-Tyr-aminoluciferin substrate (see Fig. S3 in the supplemental material) and complete inhibition of T. cruzi proteasome chymotrypsin-like activity by the remaining tested compounds, which are also inhibitors of the trypsin- and caspase-like active sites (Fig. 4; see Table S1), provided favorable evidence for the above-mentioned hypothesis. These findings are in line with those reported by Kirkman et al., who showed that both the β2 and β5 subunits of the Plasmodium falciparum proteasome were capable of hydrolysis of the fluorescence-tagged suc-Leu-Leu-Val-Tyr-AMC substrate (26). It is worth noting that bortezomib, ixazomib, and MG132 exhibited biphasic chymotrypsin-like activity dose-response curves. Taking the above into account, it is possible that these biphasic responses were a consequence of suc-Leu-Leu-Val-Tyr-aminoluciferin substrate turnover by the caspase- and/or trypsin-like active sites of the proteasome. For HTS data normalization, a control compound that can abolish T. cruzi proteasome catalytic activity would be preferred. With this in mind, and due to its highly potent inhibitory properties, bortezomib was selected as the control inhibitor.

**FIG 5:**
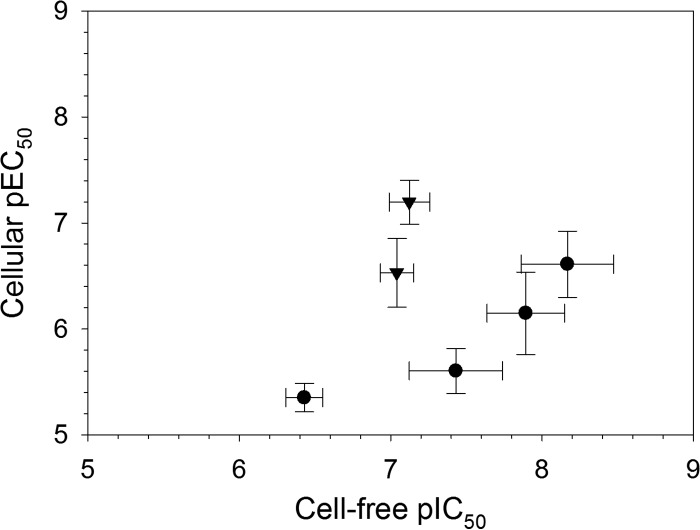
Correlation of T. cruzi proteasome pIC_50_ values obtained using cell-free and cellular assays for (from left to right) MG115, oprozomib, epoxomicin, MG132, ixazomib, and bortezomib. Circles, reversible peptide-based inhibitors; triangles, irreversible peptide-based inhibitors. Data are shown for 3 or 4 independent replicates (*n* = 3 or 4). The error bars represent SD.

**FIG 6:**
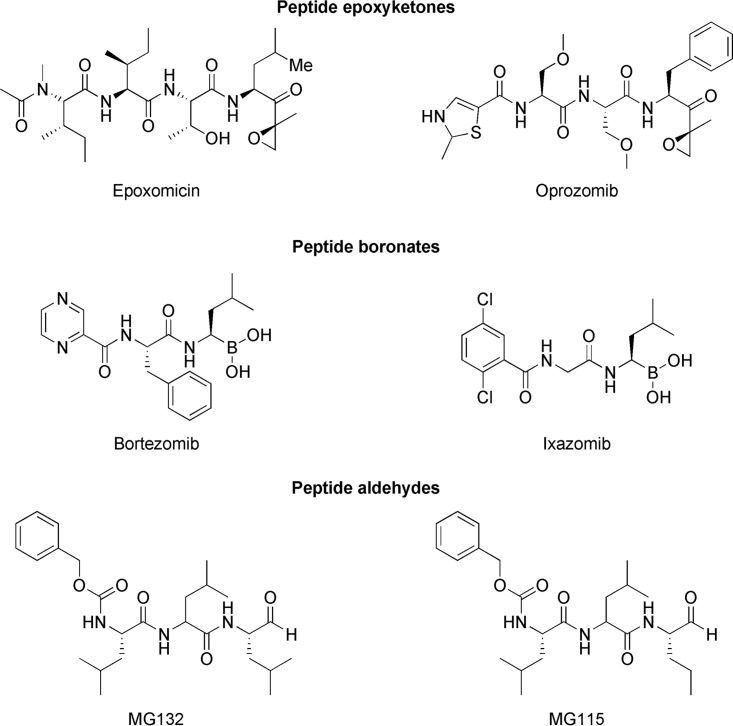
Structures of commercially available proteasome inhibitors.

Next, we compared the chymotrypsin-like pIC_50_ values (negative logarithm of half-maximal inhibitory concentration [molar]) of the commercial proteasome inhibitors with their pEC_50_ values (negative logarithm of half-maximal effective concentration [molar]) obtained using a cellular T. cruzi epimastigote viability assay (Fig. 5; see Table S1). All of the tested compounds were found to be active in the cellular assay. Epoxomicin and oprozomib exhibited equipotency between the cell-free chymotrypsin-like activity and cellular assays, while the remaining peptide-based compounds exhibited >10-fold higher potency in the cell-free versus cellular systems. Compared to a cell-free system, protein target engagement by an inhibitor in a cellular assay is dependent on a number of additional factors, including cellular penetration and retention, which are heavily influenced by the physiochemical properties of the compound, as well as the cellular substrate concentration and affinity. Therefore, it is not unusual for greater inhibitory potency to be observed in a cell-free versus a cellular assay system. The tested compounds can be clustered into peptide epoxyketone, peptide boronate, and peptide aldehyde structural classes (Fig. 6), which target the catalytic active sites of the proteasome. The peptide epoxyketones are known to be irreversible proteasome inhibitors, while the boronate and aldehyde peptide analogues exhibit their effects through reversible binding mechanisms (21, 25, 27). The lower potency in the cellular assay for the last two classes could thus be explained by a presumably high concentration of high-affinity substrates in cells (all proteins that are marked for degradation), which is something that would affect the irreversible inhibitors less.

### High-throughput screening test studies.

Following pharmacological validation of the T. cruzi proteasome luminescence-based assay, a number of test screening procedures were undertaken to evaluate the suitability of the assay in a high-throughput format. First, the sensitivity, specificity, and robustness of the platform were tested by assaying two 384-well microplates that were randomly spiked with the control compound bortezomib at concentrations that were approximately equivalent to the 30% inhibitory concentration (IC_30_), IC_50_, and IC_70_ values in this biochemical assay. The assay was found to be highly sensitive, specific, and robust, with a sensitivity of 100%, specificity of 99.4% (see Table S2 in the supplemental material), and Z factor value of 0.87. Next, a “nuisance” set of 1,027 compounds, selected to highlight common biochemical assay interference mechanisms (28), was tested. A good linear correlation was established between screening replicates of this compound set (*R*^2^ = 0.95) (see Fig. S4 in the supplemental material). However, a high number of hits that were capable of inhibiting the biochemical response by 30% or more were identified (239 compounds; hit rate = 23.3%). Further evaluation of the interference annotations for these compounds revealed that the assay appeared to be particularly sensitive to interferers with the luciferase enzyme and other mechanisms responsible for a sustained glow-like luminescence response (Table 1).

**TABLE 1:** Biochemical assay interference mechanisms identified during the screen of the nuisance compound set using the primary T. cruzi proteasome chymotrypsin-like activity luminescence-based assay and the secondary luciferase reporter counterscreen assay

Interference mechanism	Total no.^*a*^	No. of primary-screen hits^*b*^	Secondary counterscreen	
			No. of hits^*b*^	% detected^*c*^
Inhibition of luminescence-coupling system	39	37	33	89
PDELight inhibition	25	20	15	75
Luciferase inhibition	29	17	11	65
DNA binders	65	34	1	3
Redox cyclers	212	30	4	13
Zn chelators	28	5	1	20
Europium donor quenchers	26	2	0	0
Other	1,014	208	23	11

a Total number of compounds in the nuisance set annotated with the listed interference mechanism (some compounds contain primary and secondary annotations).

b Number of hits identified from nuisance set screens containing primary and/or secondary annotations for the listed interference mechanisms.

c Percentage of hits detected by the secondary counterscreen assay relative to the primary screen assay.

### High-throughput counterscreen (technology interference) assay development.

**FIG 7:**
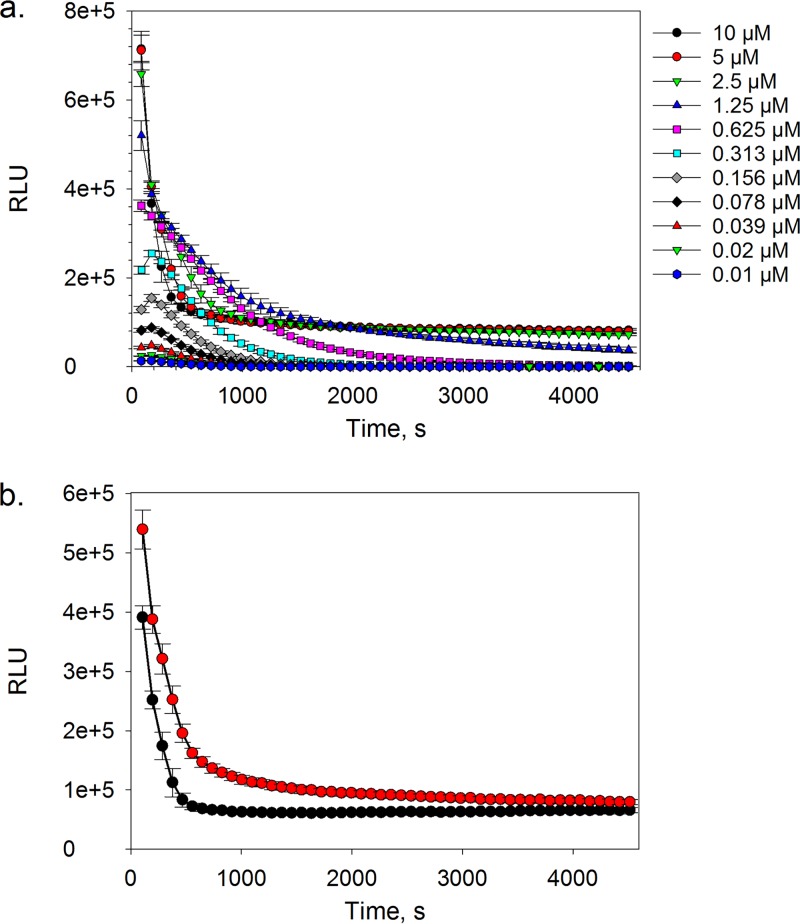
Luminescence response plotted as a function of time in the presence of various concentrations of aminoluciferin substrate (a) or in the presence (red circles) or absence (black circles) of 4 mM exogenous ATP (b). Data are shown for 4 technical replicates (*n* = 4). The error bars represent SD.

High levels of technology interference during early screening stages of the drug discovery process can translate into large numbers of false-positive hits being selected for follow-up in orthogonal assay hit confirmation studies, which are often resource intensive and have lower throughput. In an attempt to mitigate this issue, our efforts were directed toward developing a secondary high-throughput biochemical counterscreen assay in order to deconvolute luminescence technology interferers from potential T. cruzi proteasome inhibitors. By exposing the luciferase reporter component of the proteasome luminescence-based assay system to various concentrations of aminoluciferin substrate, a rapid decline in luminescence signal was observed as substrate was consumed and eventually depleted (Fig. 7a). However, it was noted that when the substrate concentration was sufficiently high (i.e., ≥2.5 μM), the rapid initial decline in signal was followed by a sustained luminescence response that lasted the duration of the experiment (i.e., 75 min). Bioluminescence is an ATP-driven process that involves the oxidation of aminoluciferin by a luciferase enzyme, resulting in the generation of a detectable photon. In order to establish a sustained glow-like luminescence response, ATP must be regenerated. Therefore, we envisaged that the luciferase reporter component of the biochemical assay was comprised of an ATP reservoir, which in the presence of sufficient aminoluciferin substrate was rapidly depleted, and the luminescence response became rate limited by ATP regeneration, thereby resulting in a sustained glow-like signal. This was rationalized by the increase in the time taken to reach a sustained luminescence response when the amount of exogenous ATP in the assay system was increased (Fig. 7b). Based on these findings and calculated Z factor values, we selected an aminoluciferin substrate concentration of 5 μM (final assay concentration) and a 60-min incubation period as desirable assay conditions.

### High-throughput counterscreen (technology interference) assay validation.

In order to validate the secondary luciferase reporter counterscreen assay, screening of the nuisance compound set was performed using the assay, and a total of 76 hits were identified (hit rate = 7.4%) using a 30% inhibition cutoff threshold. The counterscreen detected a large proportion of interferers with the luciferase enzyme and mechanisms responsible for a sustained glow-like luminescence response that were identified as hits using the primary T. cruzi proteasome chymotrypsin-like activity assay (Table 1). The inability of the counterscreen assay to detect all of the luciferase enzyme inhibitor hits could be explained by slight differences in configuration between the counterscreen and primary assays. In the case of the primary screening assay, aminoluciferin was generated *in situ* by the T. cruzi proteasome at concentrations presumably lower than those utilized in the counterscreen assay, where an excess of the substrate was required to generate a sustained response. It was therefore likely that the counterscreen assay was less sensitive to competitive inhibitors of the luciferase enzyme than the primary screening assay. It is also important to appreciate that the counterscreen assay was not designed to identify nonspecific sources of technology interference, such as DNA binders and redox cyclers, which were both prominent hits in the primary screening platform, and that some compounds in the nuisance set may be genuine inhibitors of the proteasome. However, it was envisaged that removal of technology interference at the level of the luminescence-based reporter system would sufficiently reduce false-positive hits to allow hit confirmation studies further downstream using lower-throughput orthogonal assay platforms.

### High-throughput screening of diverse compound libraries.

**FIG 8:**
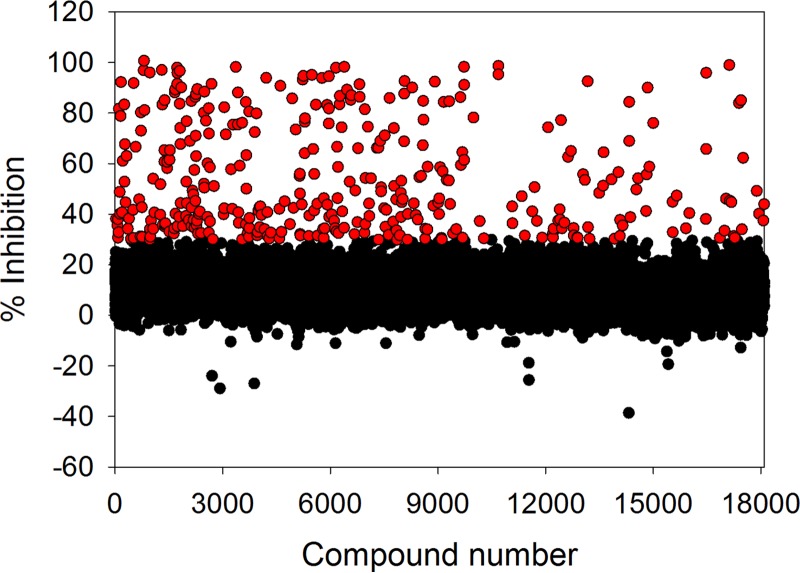
Single-point high-throughput screen of 18,098 structurally diverse compounds at a concentration of 9.4 μM using the primary T. cruzi proteasome chymotrypsin-like activity assay. The red circles represent 372 hits exhibiting ≥30% inhibition. Mean Z factor = 0.91 ± 0.03 (SD); *n* = 58.

**FIG 9:**
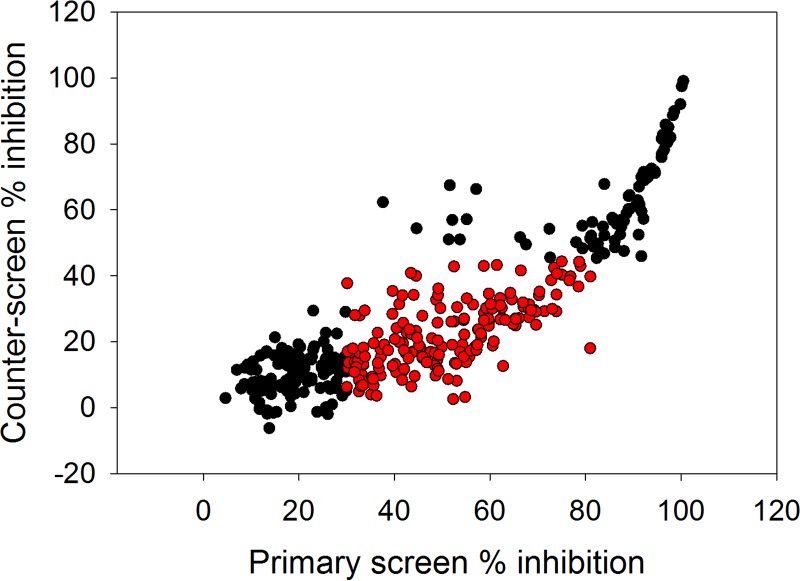
Single-point high-throughput rescreen of 372 initial hit compounds at a concentration of 9.4 μM using the primary T. cruzi proteasome chymotrypsin-like activity assay and the secondary technology interference counterscreen assay. The data represent means of two independent replicates per assay (*n* = 2). The red circles represent 180 compounds that exhibited ≥30% and <45% inhibition in the primary and secondary counterscreen assays, respectively. Primary assay mean Z factor = 0.88 ± 0.04 (SD), *n* = 4; counterscreen assay mean Z factor = 0.79 ± 0.11 (SD), *n* = 4.

**FIG 10:**
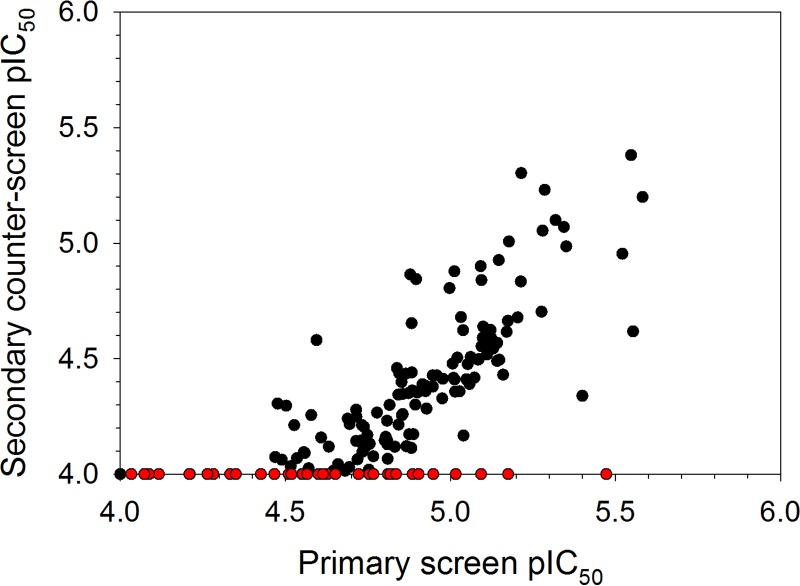
Plot of the mean pIC_50_ values (*n* = 2) of 180 hit compounds obtained using the primary T. cruzi proteasome chymotrypsin-like activity assay and the secondary counterscreen assay. The red circles represent 39 compounds that were active against the former assay (pIC_50_ ≥ 4) and inactive against the counterscreen assay (pIC_50_ < 4). Primary assay mean Z factor = 0.67 ± 0.05 (SD), *n* = 12; counterscreen assay mean Z factor = 0.77 ± 0.04 (SD), *n* = 12.

With the appropriate biochemical tools in place, we proceeded by screening two compound libraries comprising a total of 18,098 compounds covering traditional small-molecule chemical space using the primary T. cruzi proteasome chymotrypsin-like activity luminescence-based assay at a fixed compound concentration of 9.4 μM. Following this effort, we identified 372 compounds (hit rate = 2.1%) capable of inhibiting the biochemical response by 30% or more (Fig. 8). Based on our previous findings, we envisaged that the high hit rate was partially driven by technology interference at the level of the luciferase reporter system. Therefore, as an initial effort to eliminate a proportion of false-positive hits prior to downstream concentration-response assessment, the hit compounds were rescreened using the primary chymotrypsin-like activity assay, as well as the secondary counterscreen assay. By application of fixed ≥30% and <45% inhibition threshold parameters for the former and latter assays, respectively, 180 hits were identified for further evaluation (Fig. 9). Concentration-response assessment of these compounds using the above-mentioned assays was performed, and good linear correlations were obtained between the calculated pIC_50_ values from the respective assay replicates (see Fig. S5a and b in the supplemental material). Of the 180 hit compounds, 163 were found to be active in the primary T. cruzi proteasome chymotrypsin-like activity assay (i.e., pIC_50_ > 4.0) (see Fig. S5a). However, of these, only 39 compounds were found to be completely inactive (i.e., pIC_50_ ≤ 4.0) against the secondary counterscreen assay (see Fig. S6 and Table S3 in the supplemental material), with the remaining compounds exhibiting some form of technology interference (Fig. 10). Interestingly, the potency correlation between the primary chymotrypsin-like activity and counterscreen assays revealed approximately 5-fold higher pIC_50_ values for the former assay than for the counterscreen assay. This skewed relationship could be explained by the presumably lower aminoluciferin substrate concentration present in the primary screening assay than in the counterscreen assay, as described earlier, which would likely make the chymotrypsin-like activity assay more sensitive to potential interferers.

### Conclusions.

We have successfully validated a method for the production and partial purification of T. cruzi proteasomes, which we have shown to exhibit the characteristic chymotrypsin-, trypsin-, and caspase-like activities. The isolated protein material was used to adapt a commercially available glow response luminescence-based assay system into a sensitive and specific high-throughput platform aimed at identifying T. cruzi proteasome inhibitors. Interestingly, our findings suggested that the suc-Leu-Leu-Val-Tyr-aminoluciferin substrate used in this biochemical assay to probe proteasome chymotrypsin-like activity was not specific for the single type of T. cruzi proteasome active site. Instead, it appeared that a fraction of the substrate was being turned over by the trypsin- and/or caspase-like active sites of the protein. Validation of the luminescence-based biochemical assay using a nuisance compound set designed to provide an indication of types of technology interference revealed that the assay was prone to interference at the level of the luciferase reporter. To combat this issue, we developed a high-throughput secondary counterscreen assay that was sensitive to both luciferase inhibitors and inhibitors of the ATP regeneration mechanism responsible for a sustained glow-like luminescence response. We then utilized the luminescence-based T. cruzi proteasome chymotrypsin-like activity assay to screen a total of 18,098 structurally diverse compounds. Following rescreening and technology interference deconvolution using the secondary counterscreen assay, 39 hits of interest were identified. Further evaluation of these potential T. cruzi proteasome inhibitors as new chemical starting points for a Chagas’ disease drug discovery program are under way.

## MATERIALS AND METHODS

### General.

The commercially available Proteasome-Glo 3-substrate system and chymotrypsin-like assay kits (Promega; catalogue no. G8531 and G8622, respectively) were assembled according to the manufacturers’ protocols (29) unless otherwise stated. Briefly, Proteasome-Glo buffer was used to formulate the luciferin detection reagent (containing a recombinant thermostable luciferase enzyme) to approximately 0.35% (wt/vol) final assay concentration. The luciferin detection reagent was then mixed with Proteasome-Glo trypsin-, caspase-, or chymotrypsin-like reagents (comprising 15 μM Z-Leu-Arg-Arg-aminoluciferin (Z is benzyloxycarbonyl), 20 μM Z-Nle-Pro-Nle-Asp-aminoluciferin, or 20 μM suc-Leu-Leu-Val-Tyr-aminoluciferin substrate, respectively, at final assay concentrations), and the mixture was allowed to incubate at room temperature for 60 min prior to use. T. cruzi proteasome buffer comprised 50 mM Tris-HCl, pH 7.5, 10 mM sucrose, 5 mM MgCl_2_, 1 mM dithiothreitol, 2 mM ATP, 150 mM NaCl, 1 mM EDTA, and 0.05 mg/ml bovine serum albumin (BSA). T. cruzi strain Silvio X10/7 epimastigotes were maintained *in vitro* at 28°C in RTH-fetal calf serum (FCS) culture medium (RPMI 1640 supplemented with 0.4% Trypticase peptone, 0.017 M HEPES, 25 μM hemin, 10% heat-inactivated FCS). Biochemical cell-free assays were performed in an 8-μl final assay volume using 384-well white low-volume plates (Greiner; catalogue no. 784904), and cellular assays were performed in a 50-μl final assay volume using 384-well standard volume plates (Greiner; catalogue no. 781098). Luminescence was read using an EnVision 2102 Multilabel Reader (PerkinElmer, USA) with 0.2-s per well reading time, unless otherwise specified. DMSO or compound was added to the assay plates using Echo acoustic dispensers (Labcyte, USA). Reagent addition for high-throughput screening assays was performed using an Xrd-384 liquid dispenser (FluidX, United Kingdom) and a BioFill Solo/Xrd-384 8-channel resin nozzle (0.5- to 200-μl) tubing cartridge (FluidX; catalogue no. 34-1003). Data analysis was performed using SigmaPlot 12.5 software unless otherwise stated. Z factor values were calculated using the following equation: Z factor = 1 − ({3 × [1.483 × (RLU MAD Max)]} + {3 × [1.483 × (RLU MAD Min)]})/(median RLU Max − median RLU Min), where MAD is the median absolute deviation, Max is the maximum-effect control samples, and Min is the minimum-effect control samples.

### T. cruzi proteasome production and partial purification.

Mid-log-phase T. cruzi strain Silvio X10/7 cells were harvested by centrifugation (900 × *g*; 35 min). The resulting cell pellet was resuspended in Dulbecco’s phosphate-buffered saline (DPBS), and the cells were counted using a Casy Cell counter^+^ system; 2 × 10^10^ cells were pelleted in a 50-ml conical tube (900 × *g*; 15 min). The cell pellets were then inactivated by three freeze-thaw cycles and stored at −80°C. To validate biological inactivation, approximately 10% of the pellet by weight was resuspended in RTH-FCS medium and incubated at 28°C for 6 weeks. Absence of cell growth after 6 weeks was considered evidence of inactivation. For proteasome purification, T. cruzi pellets were thawed on ice, resuspended, and diluted in an equal volume of double-strength sucrose lysis buffer (100 mM Tris-HCl, pH 7.5, 500 mM sucrose, 10 mM MgCl_2_, 2 mM dithiothreitol, 4 mM ATP, 100 mM NaCl, 2 mM EDTA), followed by further dilution using single-strength sucrose lysis buffer to afford a cellular concentration of 2 × 10^9^ cells/ml. The resulting suspension was passed through a continuous-flow cell disruptor (Constant Systems Limited, United Kingdom) at 20,000 lb/in^2^ to lyse the cells. The lysate was then clarified by centrifugation at 20,000 × *g* at 4°C for 30 min, and the resulting supernatant was ultracentrifuged at 300,000 × *g* at 4°C for 120 min to form a pellet. The pellet was then resuspended using 2 ml of T. cruzi proteasome buffer (without BSA) per liter of original T. cruzi growth and solubilized by rolling at 4°C for 30 to 60 min. Insoluble material was removed by centrifugation at 20,000 × *g* at 4°C for 20 min. The sample was then passed through a 0.2-μm syringe filter and purified using a 100-ml Superose 6 gel filtration column (GE Healthcare) at a flow rate of 0.5 ml/min.

### Activity-based characterization of T. cruzi proteasome.

Four microliters of each undiluted 2-ml gel filtration fraction was incubated at room temperature with either epoxomicin (Sigma-Aldrich; catalogue no. E3652; 5 μM and 0.5% [vol/vol] DMSO final concentrations) or DMSO (0.5% [vol/vol] final assay concentration) at room temperature for 60 min. Next, 4 μl of the luciferin detection and Proteasome-Glo chymotrypsin-like reagent mixture was added to initiate the biochemical reaction. The reaction was allowed to proceed at room temperature for 20 min, after which the luminescence was read. Data were acquired from a single replicate (*n* = 1). Gel filtration fractions displaying chymotrypsin-like activity amenable to epoxomicin inhibition were pooled (see Fig. S1 in the supplemental material). Four microliters of the pooled protein material was then added to 4 μl of luciferin detection and Proteasome-Glo chymotrypsin-, trypsin-, or caspase-like reagent mixtures in the presence of either epoxomicin (5 μM and 0.5% [vol/vol] DMSO final assay concentrations) or DMSO (0.5% [vol/vol] final assay concentration). Luminescence was read immediately and then every 90 s for 75 min. Data were acquired from 6 technical replicates (*n* = 6).

### Primary assay development and kinetic-parameter determination.

Assay linearity and the optimal amount of T. cruzi proteasome for screening was determined by first serially diluting (1 in 2; CMF = 0.5) the proteasome stock solution in proteasome buffer to generate 10 additional concentrations. Next, 4 μl of each of the T. cruzi proteasome solutions was added to 4 μl of the luciferin detection and Proteasome-Glo chymotrypsin-like reagent mixture. Luminescence was read immediately and then every 90 s for 75 min. Data were acquired from 5 technical replicates (*n* = 5). To calculate the Michaelis constant (*K_m_*) and identify the optimal amount of chymotrypsin-like suc-Leu-Leu-Val-Tyr-aminoluciferin substrate for screening, the Proteasome-Glo chymotrypsin-like assay (Promega; catalogue no. G8622) luciferin detection reagent (comprising luciferase enzyme in Proteasome-Glo buffer) was prepared according to the manufacturers’ protocol (29). A 1,200 μM solution (600 μM final assay concentration) of the substrate was prepared and subsequently serially diluted (1 in 2; CMF = 0.5) using the luciferin detection reagent to generate 8 additional concentrations. Next, 4 μl of each of the luciferin detection and substrate solution mixtures was added to 4 μl of partially purified T. cruzi proteasome that was diluted 1 in 4 (CMF = 0.25) from the original stock using proteasome buffer. Luminescence was read immediately and then every 90 s for 75 min. Data were acquired from 3 technical replicates (*n* = 3). The *K_m_* was obtained by fitting all of the individual replicate data to the Michaelis-Menten equation shown below ([S] is substrate concentration):

$$v=\frac{V_{\text{max}}\left[\text{S}\right]}{K_{m}+\left[\text{S}\right]}$$

### DMSO tolerance.

Partially purified T. cruzi proteasome stock solution was diluted 1 in 4 (CMF = 0.25) using proteasome buffer, and 4 μl of the diluted solution was added to various volumes of DMSO (corresponding to 1% [vol/vol], 0.5% [vol/vol], 0.25% [vol/vol], and 0.125% [vol/vol] final assay concentrations). Next, 4 μl of the luciferin detection and Proteasome-Glo chymotrypsin-like reagent mixture was added to initiate the biochemical reaction. Luminescence was read immediately and then every 90 s for 75 min. Data were acquired from 6 technical replicates (*n* = 6).

### Cell-free pIC_50_ determinations for commercial compounds.

Partially purified T. cruzi proteasome stock solution was diluted 1 in 4 (CMF = 0.25) using proteasome buffer, and 4 μl of the diluted solution was incubated at room temperature for 60 min in various concentrations of compound (oprozomib, bortezomib, ixazomib, and MG132 [Selleckchem; catalogue no. S7049, S1013, S2180, and S2619 respectively]; MG115 [Enzo Life Sciences; catalogue no. ALX-260-091-M005]; epoxomicin [Sigma-Aldrich; catalogue no. E3652]) (12 final assay concentrations ranging from 3.09 × 10^–5^ M to 1.11 × 10^–9^ M at 1-in-3 dilution increments). Next, 4 μl of luciferin detection and Proteasome-Glo chymotrypsin-, caspase-, or trypsin-like reagent mixtures were added to initiate the biochemical reaction. The reaction was allowed to proceed at room temperature for 60 min, after which the luminescence was read. Data were acquired from 4 independent replicates (*n* = 4). Relative luminescence unit (RLU) data were normalized to percent inhibition values relative to 100% effect (DMSO, 1% [vol/vol] final assay concentration in the absence of T. cruzi proteasome enzyme) and 0% effect (DMSO, 1% [vol/vol] final assay concentration with T. cruzi proteasome enzyme) control populations using Microsoft Excel 2013 software. IC_50_ values were calculated by fitting the concentration-response data for each independent replicate separately to either a four-parameter logistic model or a seven-parameter logistic model with the bottom curve plateau parameter constrained to zero, as shown below.

The four-parameter logistic model is as follows:

$$y=\text{Min}+\frac{\text{Max}-\text{Min}}{1+{\left(\frac{x}{{\text{IC}}_{50}}\right)}^{-\text{Hillslope}}}$$

The seven-parameter logistic model is as follows:

$$y=\left(\text{Max}\_1+\left\{\frac{\text{Min}-\text{Max}\_1}{1+\left[{\left(\frac{x}{{\text{IC}}_{50}\_1}\right)}^{\text{Hillslope}\_1}\right]}\right\}\right)+\left(\text{Max}\_2-\left\{\frac{\text{Max}\_2}{1+\left[{\left(\frac{x}{{\text{IC}}_{50}\_2}\right)}^{\text{Hillslope}\_2}\right]}\right\}\right)$$

The IC_50_ parameters were then used to calculate the pIC_50_ values using the following equation, followed by a calculation of a mean pIC_50_ for each compound: pIC_50_ = {−log(IC_50_[M])}. Where biphasic concentration-response curves were observed, pIC_50_ values for the dominant curve were reported. Figures were generated by fitting the mean concentration-response data for each compound using the above-mentioned models.

### Cell-free concentration-response relationships using 20 μM and 600 μM substrate.

The Proteasome-Glo chymotrypsin-like assay (Promega; catalogue no. G8622) luciferin detection reagent (comprising luciferase enzyme in Proteasome-Glo buffer) was prepared according to the manufacturer’s protocol (29). A 1,200 μM (600 μM final assay concentration) and a 40 μM (20 μM final assay concentration) solution of Proteasome-Glo chymotrypsin-like reagent were prepared using the luciferin detection reagent as a diluent, and the mixtures were incubated at room temperature for 60 min. Partially purified stock T. cruzi proteasome solution was diluted 1 in 4 (CMF = 0.25) using proteasome buffer, and 4 μl of the diluted solution was added to various concentrations of oprozomib (Selleckchem; catalogue no. S7049) (12 final assay concentrations ranging from 3.09 × 10^–5^ M to 1.11 × 10^–9^ M at 1-in-3 dilution increments). Next, 4 μl of either 40 μM or 1,200 μM Proteasome-Glo chymotrypsin-like reagent was added to initiate the biochemical reaction. The reaction was allowed to proceed at room temperature for 60 min, after which the luminescence was read. Data were acquired from 3 independent replicates (*n* = 3) and processed as described above for the cell-free pIC_50_ determination experiments.

### Cellular pEC_50_ determinations for commercial compounds.

T. cruzi strain Silvio X10/7 epimastigotes (25 μl at 5 × 10^5^ cells/ml) were incubated at 28°C in 5% CO_2_ for 96 h in either a fixed concentration of control compound (Nifurtimox; Sigma-Aldrich; catalogue no. N3415-25MG; 4.98 × 10^–5^ M final assay concentration) or various concentrations of test compound (oprozomib, bortezomib, ixazomib, MG132, MG115, and epoxomicin; 10 final assay concentrations ranging from 4.98 × 10^–5^ M to 2.49 × 10^–9^ M at 1-in-3 dilution increments). For the cell viability readout, BacTiter-Glo microbial cell viability reagent (Promega; catalogue no. G8230) was added to each well (25 μl) and incubated at room temperature for 5 min. The plates were then sealed with clear film, and the luminescence was read using a Victor 3 (PerkinElmer, USA) or Pherastar FS (BMG Labtech, Germany) plate reader with a 0.5-s per well reading time. Data for epoxomicin were obtained from three independent replicates (*n* = 3), and data for all the remaining compounds were acquired from four independent replicates (*n* = 4). RLU data were normalized to percent inhibition values relative to 100% effect (Nifurtimox) and 0% effect (DMSO; 1% [vol/vol] final assay concentration) control populations. Normalized data for each independent replicate were fitted separately to a four-parameter logistic regression model, and pEC_50_ {i.e., −log(EC_50_[M])} values were calculated using IBDS ActivityBase 8.1.2.12 software, after which a mean pEC_50_ value for each compound was calculated.

### Secondary counterscreen assay development.

In order to identify the optimum amount of aminoluciferin substrate (Stratech; catalogue no. 13415-AAT) for screening, a 20 μM solution (10 μM final assay concentration) of substrate was prepared and subsequently serially diluted (1 in 2; CMF = 0.5) using the proteasome buffer to generate 10 additional concentrations. To test the dependence of the assay on exogenous ATP, 10 μM aminoluciferin substrate solutions (5 μM final assay concentration) were also prepared in proteasome buffer either lacking ATP (buffer A) or containing 4 mM ATP (buffer B). Next, 4 μl of each of the substrate solutions was added to 4 μl of luciferin detection reagent. Luminescence was read immediately and then every 90 s for 75 min. Data were acquired from 5 technical replicates for each experiment (*n* = 5).

### High-throughput screening test studies.

To test assay sensitivity, specificity, and suitability in a high-throughput format, partially purified stock T. cruzi proteasome solution was diluted 1 in 8 (CMF = 0.125) using proteasome buffer, and 4 μl of the diluted solution was added to two assay plates containing bortezomib (Selleckchem; catalogue no. S1013). The bortezomib was randomly distributed across a total of 22 positions per plate at final assay concentrations approximately equivalent to the IC_70_ (79 nM; DMSO, 1% [vol/vol]), IC_50_ (17 nM; DMSO, 1% [vol/vol]), and IC_30_ (4 nM; DMSO, 1% [vol/vol]) values for the compound. With the exception of the control columns, all the remaining plate wells contained DMSO only (1% [vol/vol] final assay concentration). The plates were allowed to incubate at room temperature for 60 min, after which 4 μl of Proteasome-Glo chymotrypsin-like reagent was added to initiate the biochemical reaction. The reaction was allowed to proceed at room temperature for 60 min, after which the luminescence was read. RLU values were normalized to percent inhibition values relative to 100% effect (10 μM bortezomib and DMSO, 1% [vol/vol] final assay concentrations) and 0% effect (DMSO, 1% [vol/vol] final assay concentration) control populations using Microsoft Excel 2013 software. A hit identification threshold of 30% inhibition was set, and the percent sensitivity and percent specificity were calculated using the following equations: percent sensitivity = [TP/(TP + FN)] × 100, where TP is true positive and FN is false negative, and percent specificity = [TN/(FP + TN)] × 100, where TN is true negative and FP is false positive.

### Single-point high-throughput screening.

In the case of the primary assay, 4 μl of partially purified T. cruzi proteasome that was diluted 1 in 8 (CMF = 0.125) from the original stock using proteasome buffer was added to assay plates containing either technology interference nuisance compounds (1,027 compounds; 10 μM and, for DMSO, 1% [vol/vol] final assay concentrations) or structurally diverse compounds exploring drug-like chemical space from the Dundee Drug Discovery Unit library (9,257 compounds) and Global Health Chemical Diversity Library (GHCDL) (8,841 compounds) (9.4 μM and, for DMSO, 1% [vol/vol] final assay concentrations). The plates were allowed to incubate at room temperature for 60 min, after which 4 μl of luciferin detection and Proteasome-Glo chymotrypsin-like reagent mixture was added to initiate the biochemical reaction. The reaction was allowed to proceed at room temperature for 60 min, after which the luminescence was read. For the secondary counterscreen assay, 4 μl of 10 μM aminoluciferin solution (5 μM final assay concentration) formulated in proteasome buffer was added to the compound-containing assay plates, followed by 4 μl of luciferin detection reagent to initiate the biochemical reaction. The plates were allowed to incubate at room temperature for 60 min, after which the luminescence was read. RLU values were normalized to percent inhibition values relative to 100% effect (9.4 μM bortezomib and 1% [vol/vol] DMSO final assay concentrations for the primary assay and 1% [vol/vol] DMSO in the absence of luciferase detection reagent for the secondary counterscreening assay) and 0% effect (DMSO, 1% [vol/vol] final assay concentration) control populations. For the nuisance compound set, primary assay screening data were acquired from 2 independent replicates (*n* = 2), while the secondary assay counterscreen data were acquired from a single replicate (*n* = 1). For initial single-point high-throughput screening of the two diversity compound sets, data were acquired from a single replicate (*n* = 1) and follow-up screening data on identified hits using both the primary and secondary counterscreen assays were acquired for two independent replicates (*n* = 2). Compounds exhibiting ≥30% and ≥45% inhibition in the primary and secondary counterscreen assays, respectively, were identified as hits. Data were processed using IBDS ActivityBase 8.1.2.12 and Dotmatics Limited Vortex v2017.08.69598-59-s software.

### Hit compound cell-free pIC_50_ determinations.

Primary screen and secondary counterscreen biochemical assays were performed as described above for the single-point high-throughput screening experiments using assay plates containing various concentrations of compound (10 final assay concentrations ranging from 9.90 × 10^–5^ M to 5.52 × 10^–9^ M at 1-in-3 dilution increments). Data were acquired from 2 independent replicates (*n* = 2) for both the primary and counterscreen assays and were subsequently processed using IBDS ActivityBase 8.1.2.12 and Dotmatics Limited Vortex v2017.08.69598-59-s software. pIC_50_ values were determined by fitting the data to a four-parameter logistic model.

### Ancillary information.

The supplemental material includes fraction testing of the T. cruzi proteasome, chymotrypsin-like activity in the presence of different concentrations of DMSO, cell-free pIC_50_ and cellular pEC_50_ values for commercial inhibitors, concentration-response curves for oprozomib in the presence of low and high substrate, primary biochemical assay sensitivity and specificity calculations, a high-throughput screen of nuisance set compounds, pIC_50_ correlation plots for primary and secondary counterscreen assay replicates, structures of 39 hit compounds, and primary assay pIC_50_ values for the 39 hit compounds.

## Supplementary Material

Supplemental file 1
