# Supplementary material for: Identification of Novel Trypanosoma cruzi Proteasome Inhibitors Using a Luminescence-Based High-Throughput Screening Assay
Source: Antimicrob Agents Chemother. 2019 Aug 23;63(9):e00309-19. doi: 10.1128/AAC.00309-19 (PMC6709497; doi:10.1128/AAC.00309-19)
Supplement: Supplemental file 1 [file AAC.00309-19-s0001.pdf]

Identification of novel Trypanosoma cruzi proteasome inhibitors using a luminescence-based high-throughput screening assay.

Supporting Information.

Filip Zmuda[†], *Lalitha Sastry*[†], *Sharon M. Shepherd*[‡], *Deuan Jones*[†], *Alison Scott*[‡], *Peter D. Craggs*[⊥], *Alvaro Cortes*[⊥], *David. W. Gray**[†], *Leah S. Torrie* [†], *Manu De Rycker**[†]

[†]Drug Discovery Unit, Wellcome Centre for Anti-Infectives Research, School of Life Sciences, University of Dundee, Dow Street, Dundee DD1 5EH, United Kingdom.

[‡]Protein Production Team, Wellcome Centre for Anti-Infectives Research, School of Life Sciences, University of Dundee, Dow Street, Dundee DD1 5EH, United Kingdom.

[⊥]Screening Compound Profiling and Mechanistic Biology, Platform Technology and Science, GlasoSmithKline, Gunnelswood Road, Stevenage SG1 2NY, United Kingdom.

Table of Contents

1. Fraction testing of *T. cruzi* proteasome material post gel-filtration. S2
2. Chymotrypsin-like activity of the *T. cruzi* proteasome in the presence of different concentrations of DMSO. S2
3. Cell-free pIC_{50} and cellular pEC_{50} values for commercial inhibitors. S3
4. Concentration-response curves for oprozomib in the presence of 20 μM and 600 μM substrate. S4
5. Primary biochemical assay sensitivity and specificity calculations. S4
6. High-throughput screen of the ‘Nuisance’ validation compound set. S4
7. pIC_{50} correlation plots for primary and secondary counter-screen assay replicates. S5
8. Structures of 39 hit compounds (**1**–**39**) that were active in the primary assay and inactive in the secondary counter-screen assay. S6-S7
9. Primary assay pIC_{50} values for compounds **1**–**39**. S8

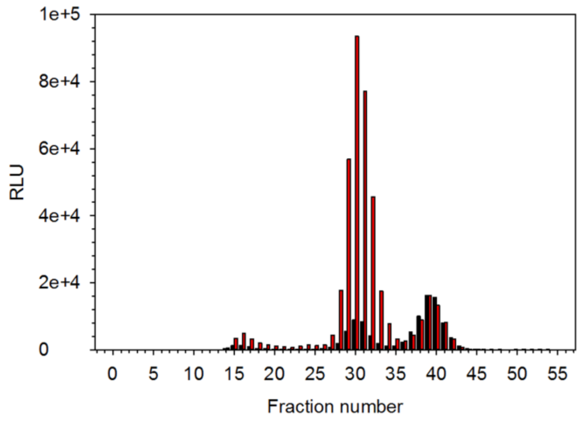

Figure S1. Fraction testing of *T. cruzi* proteasome material post gel-filtration purification in the presence (black bars) and absence (red bars) of 5 μM epoxomicin. DMSO = dimethyl sulfoxide. Data acquired from a single experiment.

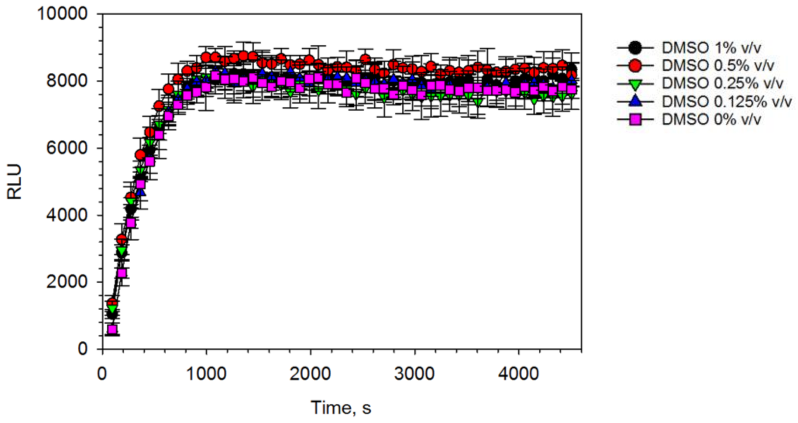

Figure S2. Chymotrypsin-like activity of the *T. cruzi* proteasome in the presence of different concentrations of DMSO. Data were acquired from 6 technical replicates (n = 6). Error bars represent ± SD.

Table S1. Cell-free chymotrypsin-like activity pIC_{50} and cellular pEC_{50} values for a panel of commercially available proteasome inhibitors.

**Compound**	**Cell-free pIC_{50}[a] ± SD, M**			**Cellular pEC_{50}[b] ± SD, M**
	Chymotrypsin-like	Trypsin-like	Caspase-like	
Oprozomib	7.04 ± 0.11	n.a.[c]	n.a.[c]	6.53 ± 0.32
Epoxomicin	7.12 ± 0.13	6.10 ± 0.06	5.85 ± 0.05	7.20 ± 0.21
MG115	6.43 ± 0.12	4.87 ± 0.09	4.91 ± 0.10	5.36 ± 0.13
MG132	7.43 ± 0.31	4.88 ± 0.14	6.01 ± 0.09	5.61 ± 0.21
Ixazomib	7.89 ± 0.26	5.51 ± 0.07	7.49 ± 0.17	6.15 ± 0.39
Bortezomib	8.17 ± 0.30	6.48 ± 0.08	7.16 ± 0.13	6.62 ± 0.31

[a]Cell-free chymotrypsin-, trypsin-, and caspase-like activity pIC_{50} values were acquired for the *T. cruzi* proteasome using the biochemical luminescence assay. Parameters calculated from four independent replicates (n = 4). [b]Cellular pEC_{50} values were acquired using a *T. cruzi* epimastigote assay. Parameters for epoxomicin were calculated from three independent replicates (n = 3) and parameters for all remaining compounds were calculated from four independent replicates (n = 4). [c]n.a. = not active.

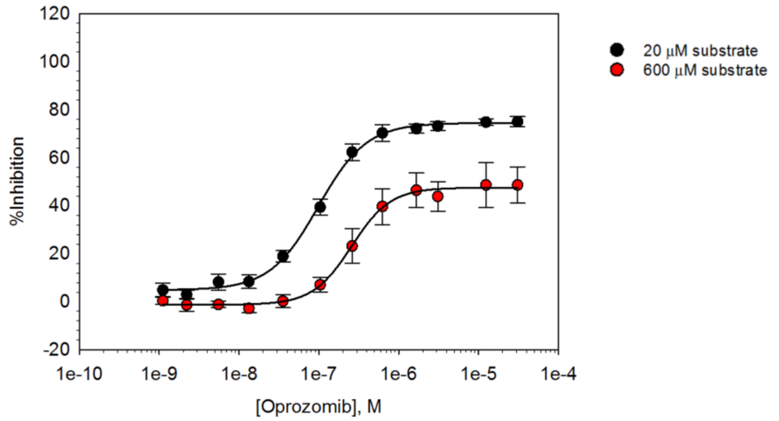

Figure S3. Cell-free *T. cruzi* proteasome chymotrypsin-like activity concentration-response curves for oprozomib obtained in the presence of 20 µM and 600 µM suc-Leu-Leu-Val-Tyr-aminoluciferin substrate. Data shown for three independent replicates (n = 3). Error bars represent ± SD.

Table S2. Identified hits from the *T. cruzi* proteasome luminescence assay during a high-throughput screening test using the control compound bortezomib at concentrations that were approximately equivalent to the IC_{30}, IC_{50}, and IC_{70} values. Compounds exhibiting % inhibition ≥30% were identified as hits.

Test Positive	TP	FP	Total test positives
	44	4	48
Test Negative	FN	TN	Total test negatives
	0	660	660
	Total positive	Total negative	Total sample
	44	664	708
**Sensitivity**		100%	
**Specificity**		99.40%	

TP = true positive; FP = false positive; FN = false negative; TN = true negative.

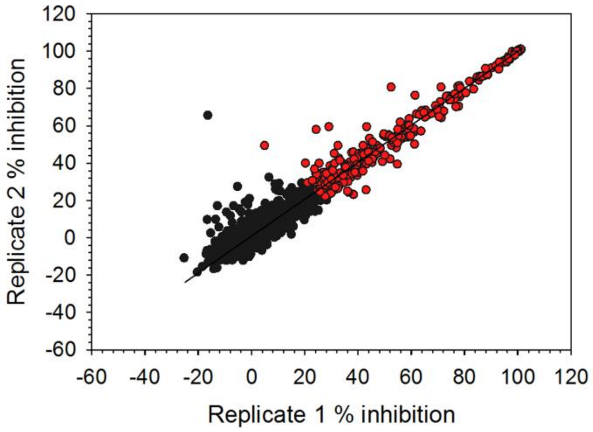

Figure S4. High-throughput screen of the ‘Nuisance’ validation compound set using the *T. cruzi* proteasome chymotrypsin-like activity biochemical assay. Data shown for 2 independent replicates. Red data points represent hits with ≥30% inhibition. Linear regression R^2 = 0.95; and Z factor ≥0.86.

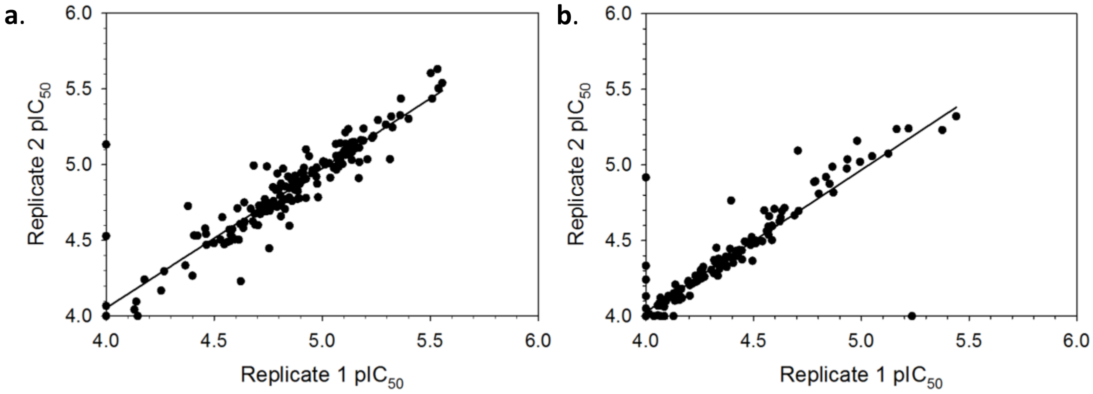

Figure 5S. Correlation of pIC_{50} values for 180 hit compounds between two experimental replicates acquired using the *T. cruzi* proteasome chymotrypsin-like activity primary screening assay (a) (R^2 = 0.89) and the technology interference counter-screen assay (b) (R^2 = 0.84).

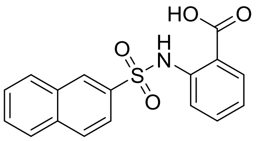

1

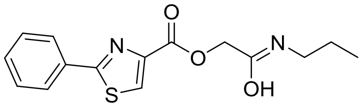

2

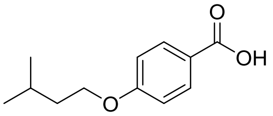

3

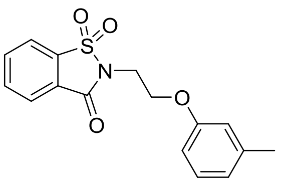

4

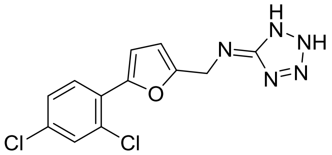

5

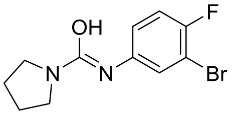

6

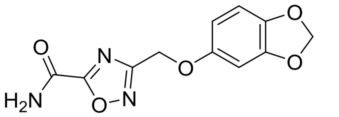

7

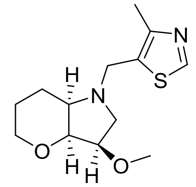

8

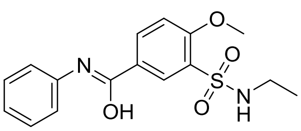

9

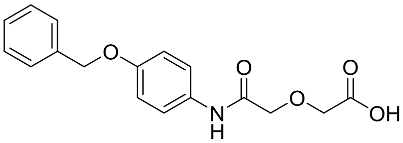

10

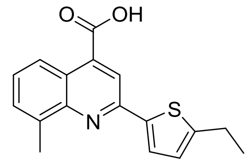

11

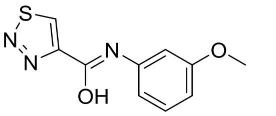

12

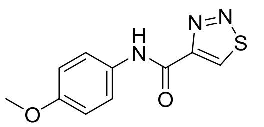

13

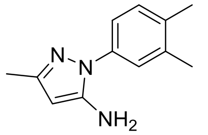

14

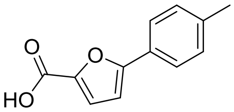

15

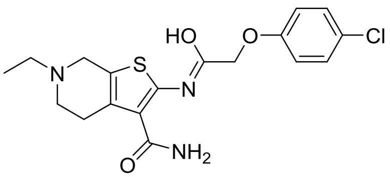

16

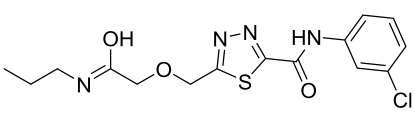

17

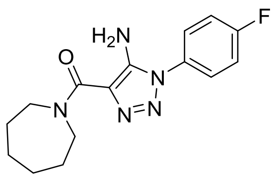

18

66

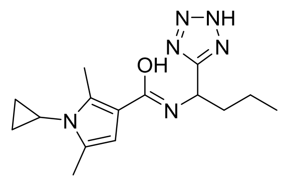

19

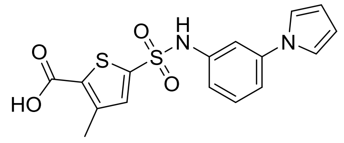

20

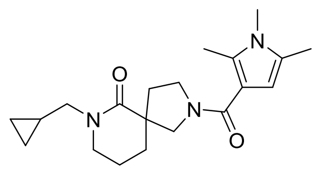

21

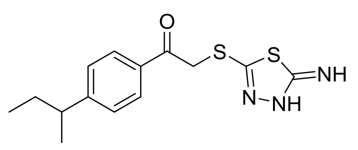

22

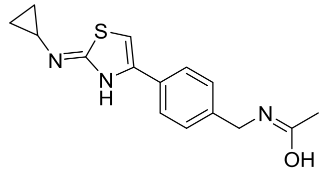

23

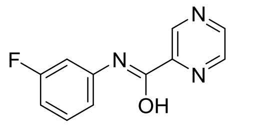

24

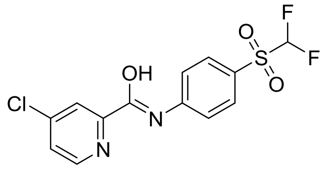

25

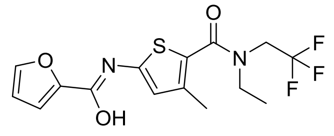

26

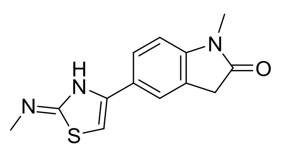

27

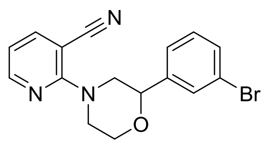

28

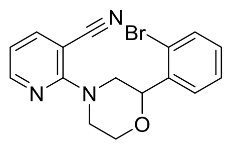

29

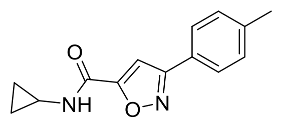

30

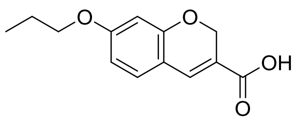

31

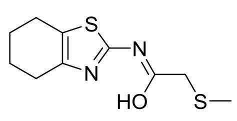

32

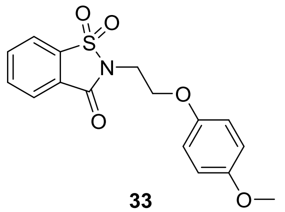

33

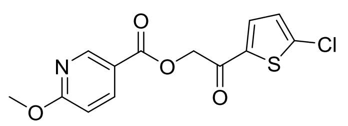

34

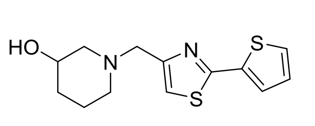

35

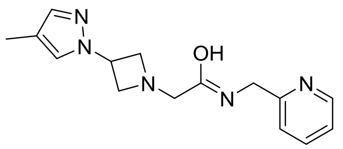

36

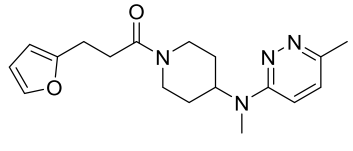

37

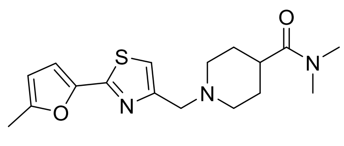

38

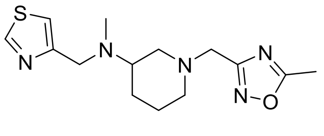

39

Figure S6. Structures of the 39 hit compounds (**1**–**39**) that were found to be active in the primary *T. cruzi* proteasome chymotrypsin-like activity assay (i.e. $pIC_{50} \geq 4.0$) and inactive in the secondary technology interference counter-screen assay (i.e. $pIC_{50} < 4.0$).

Table S3. pIC_{50} values for compounds **1–39** acquired using the primary *T. cruzi* proteasome chymotrypsin-like activity assays. Data shown is from two independent replicates (n = 2).

Compound	Replicate 1 pIC_{50}	Replicate 2 pIC_{50}
**1**	4.58	4.52
**2**	4.46	4.47
**3**	4.40	4.27
**4**	4.87	4.90
**5**	4.62	4.23
**6**	4.95	4.94
**7**	5.05	4.98
**8**	5.07	4.97
**9**	4.64	4.62
**10**	4.68	4.62
**11**	4.14	4.09
**12**	4.59	4.51
**13**	4.77	4.85
**14**	4.38	4.73
**15**	4.55	4.47
**16**	5.11	5.07
**17**	5.51	5.44
**18**	4.80	4.73
**19**	4.25	4.17
**20**	4.78	4.72
**21**	4.00	4.07
**22**	4.00	5.13
**23**	4.27	4.30
**24**	4.18	4.24
**25**	4.91	4.90
**26**	4.00	4.53
**27**	4.13	4.04
**28**	4.85	4.60
**29**	4.89	4.77
**30**	5.31	5.04
**31**	4.70	4.60
**32**	4.75	4.45
**33**	4.80	4.84
**34**	4.15	4.00
**35**	4.62	4.61
**36**	4.83	4.71
**37**	4.89	4.78
**38**	4.37	4.33
**39**	4.46	4.58
